# Magnitude and outcomes of road traffic accidents at Hospitals in Wolaita Zone, SNNPR, Ethiopia

**DOI:** 10.1186/s13104-015-1094-z

**Published:** 2015-04-09

**Authors:** Feleke Hailemichael, Mohammed Suleiman, Wondimagegn Pauolos

**Affiliations:** Wolaita Sodo University School of Public Health, P.O. Box, 138, Wolaita Sodo, Ethiopia; Wolaita Sodo University Department of Anesthesia, Wolaita, Ethiopia

**Keywords:** Road traffic accident, Trauma

## Abstract

**Background:**

A Road traffic accident is an incident on a way or street open to public traffic, resulting in one or more persons being killed or injured, and involving at least one moving vehicle.

**Methods:**

The aim of this study is to assess magnitude and outcome of road traffic accidents among trauma victims at hospitals in Wolaita zone. A cross sectional hospital based study design using retrospective chart review was conducted from March 5th to March 25th, 2014. Simple random sampling technique was applied to identify sample population. The data was entered in to Epi info version 3.5.1 and transferred to SPSS version 16 for further analysis.

**Results:**

A total of 384 trauma victims were incorporated in the study of which 240 (62.5%) were due to road traffic accidents. The majority of patients were male 298 (77.6%) and most commonly aged between 20–29 (35.42%). The principal outcome of injury was more commonly lower extremity (182 patients, 47.4%), compared to upper extremity (126 patients, 32.8%).

**Conclusion:**

Of all trauma patient presenting to hospitals (62.5%) are the result of road traffic accident. Hence, the provision of tailored messages to all members of the community regarding knowledge and practices of road safety measures like appropriate use of pavements by pedestrians and avoiding risky driving behaviors. Besides this make use of compulsory motorcycle helmets would appear to be a very important intervention

## Background

Accident is an incident, occurring suddenly, unpredictably and unintentionally under unforeseen conditions [1]. Road Traffic Accident (RTA) can be defined as an accident that occurs on a way or street open to public traffic, resulting in one or more persons being killed or injured, and involving at least one moving vehicle. Accordingly, RTAs are collisions between vehicles, between vehicles and pedestrians, between vehicles and animals, or between vehicles and geographical or architectural obstacles [2].

Road traffic accidents have become a huge global public health and development problem killing nearly 1.2 million people a year and injuring or disabling between 20–50 million people worldwide; thus making the loss of 518 billion US $ globally [3]. The report written by WHO in 2013 showed that more than 1.24 million people die every year as a result of road traffic injuries, making it “the eighth leading cause of death globally, and the leading cause of death for young people aged 15–29”. Based on current trends, it is projected to be the fifth leading cause of death globally by 2030 [4].

In the developing world, the improved life expectancy together with industrialization and urbanization are putting heavy pressure on the transport system in general and on road system in particular [5]. In addition, when compared to the developed nations, causes for high burden in road traffic-related deaths and injuries in developing countries are primarily due to an increase in motor vehicle numbers, poor enforcement of traffic safety regulations, inadequacy of public health infrastructure, and poor access to health services, etc. [6]. RTAs have been the principal causes of fatality and disability in African countries mainly among those aged 5–29 years. Every day in Africa, about 2,400 individuals die from injuries of which the leading cause of injury is due to road traffic crash. In the African countries deaths from road traffic injuries are 40% higher than in all other low and middle income countries and 50% higher than the world average [7-9].

Ethiopia, a developing country in Africa, has witnessed a number of the most risky roads in the world and has followed to overtake a determined road spreading out guiding principle in the past 15 years [10]. The Ethiopian National Road Safety Coordination Office cites a road crash fatality rate of 114 deaths per 10 000 vehicles per year but the actual figure may be higher due to an improper reporting system [10,11]. This compares to a mortality rate of one death per 10 000 vehicles per year in the United Kingdom and an average mortality rate of 60 per 10 000 vehicles across 39 sub-Saharan African countries [10].

The intention of this study is to assess magnitude of road traffic accident and its resultant injuries among trauma victims from the data of Trauma care and Victim Information System at hospitals in Wolaita zone, southern Ethiopia. To the level of our knowledge such a study has never been conducted in the Wolaita zone, thereby recognizing the need to establish baseline information so that it can subsequently be used by local road safety measures and stake holders to identify priorities and devise targeted interventions and preventive measures to improve road safety among road users. In addition to this, it can also be used as making a sole input to the literature.

## Methods

### Study settings

The study employed a cross-sectional study design. Retrospective chart review was performed from March 5th to March 25th, 2014 at Hospitals in Wolaita Zone. The administrative city of the Zone is Wolaita Sodo, which is located 327 km from Addis Ababa There are three hospitals in the zone, one governmental and two nongovernmental by ownership.

### Source population

The source population was all charts of trauma victims that visited the hospitals in Wolaita Zone.

### Study population

Study populations included in the study was selected charts of trauma victims that were visited, Hospitals in Wolaita Zone from September 1st, 2012 to September 1st, 2013.

### Sample size and sampling technique

The number of charts included for the study was estimated by applying a single population proportion formula with the following assumptions: α = the risk of rejecting the null hypothesis (0.05), d = degree of precision or margin of error (0.05), Z = the standard score corresponding to a 95% confidence interval and p = 50% (the proportion of road traffic accident). Accordingly the final sample size was 384. By using simple random sampling technique, selected cards were identified and traced using registration number.

### Data collection instruments and procedure

Pretested checklist or formats prepared in English were used as a data collection instrument. During data collection, two medical record officers and ten nurse diploma holders were involved. Supervisors crosschecked for completeness and consistency of collected data on a daily basis. During the data collection, record keepers sorted out all the trauma cases from log books and medical records. Data collectors traced and collected data from randomly identified charts of trauma cases using checklist.

### Data analysis

Data was collected and checked for completeness and accuracy manually, then coded and entered in to Epi data version 3.5.3 computer software by investigators. The data were subsequently transferred to SPSS version 16 computer program for analysis. The result was presented in narrative and tables.

### Ethical consideration

Approval was obtained from Wolaita Sodo University, College of Health Sciences and Medicine, Research Ethical Review Committee. The advantages and purposes of the study were explained to staff members of the hospitals. Then, for retrieval of individual record and confidentiality of information a written consent was given to the record office of the hospitals. After completion of data collection, medical records were returned back to their original place properly.

## Results

### Socio-demographic characteristics

A total of 384 trauma victims were included in the study. Of all victims visited the hospitals 298 (77.6%) were male and 86 (22.4%) were female. The mean age of the victims was 25.5. The highest numbers of victims (35.42%) were aged between 20–29 followed by age 10–19 years (26.04%). Concerning ethnicity of the study population, Wolaita was the most common at 173 (45%) followed by Oromo at 44 (11.4%). RTAs presenting to hospital more commonly occurred in urban areas; in contrast non road traffic accident was the highest on clients from rural areas. See Table 1.

**Table 1:** **Socio-demographic characteristics of trauma victims from September 1, 2012 to September 1, 2013, at hospitals in Wolaita Zone, SNNPR, Ethiopia**

**Variables**	**Accidents**		**Total**
	**Road trafic accident**	**Non road trafic accident**	
Sex			
Male	185 (48.18%)	113 (29.43%)	298 (77.6%)
Female	55 (14.32%)	31 (8.07%)	86 (22.4%)
Age			
<10	19 (4.94%)	6 (1.57%)	25 (6.51%)
10-19	55 (14.32%)	45 (11.72%)	100 (26.04%)
20-29	88 (22.92%)	48 (12.50%)	136 (35.42%)
30-39	47 (12.24%)	22 (5.74%)	69 (17.98%)
40-49	21 (5.5%)	13 (3.35%)	34 (8.85%)
50-59	3 (0.78%)	3 (0.78%)	6 (1.56%%)
>60	7 (1.82%)	7 (1.82%)	14 (3.64%)
Ethnicity			
Wolaita	117 (30.4%)	56 (14.6%)	173 (45%)
Gammo	13 (3.3%)	12 (3.2%)	25 (6.5%)
Amhara	6 (1.6%)	2 (0.7%)	8 (2.8%)
Hadiya	15 (4%)	9 (2.3%)	24 (6.3%)
Alaba	3 (0.7%)	3 (0.7%)	6 (1.4%)
Oromo	22 (5.7%)	22 (5.7%)	44 (11.4%)
Kembata	13 (3.4%)	14 (3.6%)	27 (7%)
Others*	51 (13.3%)	26 (6.7%)	77 (20%)
Place of residence			
Urban	148 (38.5%)	67 (17.7%)	215 (56%)
Rural	92 (24%)	77 (20%)	169 (44%)

*Gurage, Sidama.

### Causes of injury and duration of pre-hospital phases

RTAs were the leading cause of injury, accounting for 240 (62.5%) of all trauma victims followed by falling accident 20.8% and personal violence 9.6%. When we come to pre-hospital phase, 301 (78.4%) of trauma victims were presented to the hospitals within 24 hours to one week of the injury followed by less than 24 hours 41 (10.7%). See Table 2.

**Table 2:** **Causes of injury and duration of pre-hospital phases among trauma victims from September 1, 2012 to September 1, 2013, at hospitals in Wolaita Zone, SNNPR, Ethiopia**

**Causes of injury**		
**Variable category**	**Frequency**	**Percent (%)**
Road traffic accident	240	62.5
Falling accident	80	20.8
Personal violence	37	9.6
Gun shoot	17	4.4
Burn	10	2.6
**Duration of pre-hospital phase**		
**Variable category**	**Frequency**	**Percent (%)**
Less than 24 hours	41	10.7
24 hours to one weeks	301	78.4
one week to two weeks	33	8.6
Three weeks to a month	6	1.5
Above a month	3	0.8

### Vehicle type that caused road traffic accident and Patient’s role at the time of injury

Among 240 road traffic accidents, 75 (31.2%) were due to motor cycle crash followed by 52 (21.7%) due to Isuzu and 34 (14.2%) due to Bajaj related accident. Out of 240 victims of road traffic injury, 110 (45.8%) were passengers, 81 (33.8%) were pedestrians and 45 (18.8%) were drivers. See Table 3.

**Table 3:** **Vehicle types that caused road traffic accident and patient role at the time of road traffic injury from September 1, 2012 to September 1, 2013, at hospitals in Wolaita Zone, SNNPR, Ethiopia**

**Vehicle type that caused road traffic accident**		
**Vehicle type**	**Frequency**	**Percent (%)**
Vehicle type		
Isuzu	52	21.7
Motor cycle	75	31.2
Public Bus	23	9.6
Minibus	26	10.8
Bajaj	34	14.2
Others**	30	12.5
**Patient role at the time of road traffic injury**		
**Role**	**Frequency**	**Percent (%)**
Pedestrian	81	33.8
Passenger	110	45.8
Driver	45	18.8
Assistant driver	4	1.7
Total	240	100.0

**Pickup, Cart.

### Diagnoses, length of hospital stay (LOS) and patients’ outcome

Of 384 trauma victims, 212 (55%) stayed in hospital for less than one week, 123 (32%) stayed in hospital from two to three weeks, 18 (4.7%) stayed in hospital for three weeks to one month, and the remaining 31 (8.3%) stayed for more than one month.

Lower extremity represented majority of injury site 182 (47.4) followed by upper extremity injury 126 (32.8). Of all victims reaching hospital, 23 (6%) died, 48 (12.5%) survived with long term disability on discharge and 313 (81.5%) survived without long term disability on discharge. See Table 4.

**Table 4:** **Diagnosis and Outcome of the trauma victims from September 1, 2012 to September 1, 2013, at hospitals in Wolaita Zone, SNNPR, Ethiopia**

**Variables**	**Frequency**	**Percent (%)**
Patients condition on arrival or discharge		
Survived without long term disability	313	81.5
Survived with long term disability	48	12.5
Died on arrival	13	3.4
Died on discharge	10	2.6
Total	384	100
**Diagnosis**		
Upper extremity injury	126	32.8
Trunk injury	76	19.8
Lower extremity injury	182	47.4
Total	384	100

## Discussion

In this study it is found that RTAs are the most common cause of traumatic injury, followed by falling accidents and personal violence. Studies conducted in Tikur Anbessa Hospital, Addis Ababa [12] and in North Gondar zone [13] reported road traffic accident at the top with different trend, but followed by interpersonal violence and falling accidents respectively.

The current study revealed that 156 (40.64%) of the victims were in age group between 20–49 years which is consistent with other similar studies [12-14]. This shows that a large amount of sufferers are people of most economically active age group that subsequently leads an economic lost both to the family and the nation. The results of this study also found that males were much more likely to suffer from injuries than females. This is likely due to the nature of work exposing, majority of males on urban streets or the increased level of participation in high-risk activities among male individuals.

In line with other studies [15,16] majority of traumatic victims were passengers followed by pedestrians, but in another studies it was reported that pedestrians were majority of cases and followed by passengers [17-19]. The predominance of traumatic injury by passenger may be related with greater use of public transport by general population, risky driving behavior and the driving skill of their operators. The frequency of pedestrian injury in this study may be a sign of low public consciousness on road use, shortage of pedestrian facilities in road design and poor practice of road safety measures by the general population.

In the present study, it is found that greater part of the victims visited the hospitals within 24 hours to one week of the injury followed by less than 24 hours. Similarly, a study conducted in a rural hospital in Ghana reported almost half of the cases were presented within a week after the injury [20]. However, a study done in Tehran city of Iran showed that average pre-hospital time interval was 7.1 min which is much shorter than the result of our study [21]. The difference in duration of pre-hospital interval time across the country may be due to variation in infrastructure of the city including transportation system, road design and availability of nearby hospitals.

It was found that motorcycles were responsible for the majority of road traffic crashes that is consistent with the study conducted on motorcycle injuries as an emerging public health problem in Mwanza City, north-western Tanzania [22]. In contrast, it is lower than the study conducted on injury characteristics and outcome of road traffic crash victims at Buganda Medical Centre in Northwestern Tanzania [16]. In this study LOS for the majority of trauma victims was less than one week. This is consistent with study conducted at hospitals in Tehran city [23-25]. In contrast, the average LOS for road traffic injuries (RTIs) in the current study was lower than the average LOS for RTIs reported in Trinidad and Tobago on a contemporary analysis of road traffic crashes, fatalities and injuries [26]. It is difficult to compare length of stay between countries, due to differences by the care given in organization of trauma care and variations in patterns of injury.

The finding of this study also showed that of all deaths, the majority of victims died during the pre-hospital phase and were brought to the hospital for medical evidence. This agrees with other similar studies [27,28]. Findings in this study should be interpreted in the light of the inherent limitations of the study. We could not take more information on certain risk factors like educational status of victims because of lack of available data from the records; since this was a hospital based cross-sectional study.

## Conclusion and recommendation

### Conclusion

The incidence of trauma caused by RTA was the highest cause of trauma related admission (62.5%). RTA victims were predominantly males and people aged 20–49 years. The majority of them are from urban areas. Patients from rural areas more commonly presented with non-RTA trauma. Of all RTAs motor vehicle crash was the primary causes of injury. Passengers and pedestrians were the most commonly affected victims. The majority of the victims visited the hospitals from 24 hours to one week following the injury, representing a significant delay in presentation. Of all the trauma fatalities, death most commonly occurred during the pre-hospital phase.

### Recommendation

This study provides baseline information from hospitals in the Wolaita zone, which could help with possible interventions regarding RTA. The provision of tailored messages to all members of the community regarding knowledge and practices of road safety measures like appropriate use of pavements by pedestrians and avoiding risky driving behaviors. Besides this make use of compulsory motorcycle helmets would appear to be a very important intervention to decrease road traffic accidents. It is important to establish and strengthen advanced pre-hospital care and an effective ambulance system for transportation of patients to hospitals as soon as possible. Promoting rehabilitation services for all trauma victims and good medical recording system also indicated. Further research should be conducted in order to explore the risk factors that hinder and/or aggravate the frequency of RTAs in the community.
